# Serum Cortisol, 25 (OH)D, and Cardiovascular Risk Factors in Patients with Type 2 Diabetes Mellitus

**DOI:** 10.1155/2022/5680170

**Published:** 2022-06-18

**Authors:** Yuxi Jin, Dandan Wei, Pengling Liu, Fei Chen, Rongrong Li, Jinyu Zhang, Ruyi Zhang, Zuoxiang Liu, Wenqian Huo, Linlin Li, Chongjian Wang, Jinbao Ban, Zhenxing Mao

**Affiliations:** ^1^Department of Thoracic Surgery, The First Affiliated Hospital of Zhengzhou University, Zhengzhou 450052, China; ^2^Department of Epidemiology and Biostatistics, College of Public Health, Zhengzhou University, Zhengzhou, Henan, China; ^3^Department of Oncology, the First Affiliated Hospital of Xinjiang Medical University, Urumqi Municipality, Xinjiang, China

## Abstract

**Background and Aims:**

The effects of cortisol on cardiovascular diseases (CVD) and CVD risk are unknown, especially in patients with type 2 diabetes mellitus (T2DM). Furthermore, it is unclear whether 25 (OH)D can alter the associations of cortisol with CVD and CVD risk factors. Thus, the present study was to investigate the associations of serum cortisol with CVD and CVD risk factors and whether 25 (OH)D altered these associations among patients with T2DM. *Materials and methods*. A total of 762 patients diagnosed with T2DM were recruited. The levels of serum cortisol and 25 (OH)D were measured with a liquid chromatography-tandem mass spectrometry. Logistic regression and linear regression were used to assess the association of cortisol with CVD and multiple cardiovascular risk factors. Modification analyses were performed to identify whether 25 (OH)D altered the above associations.

**Results:**

A 1 SD increase in cortisol was associated with a higher prevalence of stroke (odds ratio (OR): 1.25, 95% confidence interval (CI): 1.05, 1.50). Elevated cortisol was associated with related cardiovascular risk factors, including deceased *ß* cell function, high-density lipoprotein-cholesterol (HDL-C), and fasting insulin, as well as increased triglycerides (TG), low-density lipoprotein-cholesterol (LDL-C), fasting plasma glucose (FPG), and glycated hemoglobin (HbA1c). In addition, modification analyses suggested that the associations of cortisol with *ß* cell function, fasting insulin, FPG, and HbA1c were modified by 25 (OH)D.

**Conclusions:**

Serum cortisol was associated with the prevalence of stroke and cardiovascular risk factors, and the associations of cortisol with cardiovascular risk factors were moderated by 25 (OH)D, suggesting that T2DM patients with exposure to lower 25 (OH)D levels and higher cortisol levels were more susceptible to have higher cardiovascular risk factors.

## 1. Introduction

Cardiovascular diseases (CVD), together with its related morbidity and mortality, pose significant personal, societal, and economic burdens worldwide. The epidemic of CVD in China is emerging as a result of lifestyle changes, urbanization, and the accelerated process of ageing. The incidence of CVD is continuously increasing and will remain an upward trend in the next decade. CVD is the leading cause of death among urban and rural residents in China, and epidemiological studies found that 44.8% of deaths in rural areas and 41.9% of deaths in urban areas were caused by CVD in 2014 [1]. In addition, China has the largest number of people with type 2 diabetes mellitus (T2DM), accounting for more than a quarter of the total number of adult diabetes patients in the world [2, 3]. People with T2DM have about a two-fold increased risk of CVD compared to healthy people [4]. The risk of coronary artery disease (CHD) increases in patients with diabetes by 11% for each 1% increment in glycated hemoglobin (HbA1c) greater than 6.5% [5]. Therefore, the risk factors of CVD in patients with T2DM deserve our attention. Although existing researches explored a lot of factors about the incidence of CVD, the direct evidence involved in CVD risk factors in T2DM patients is scant.

The association of chronic stress and CVD has been suggested in both animal and human studies [6, 7]. Cortisol has been considered as a biomarker of chronic stress, suggesting long-term increase in response to chronic stressors [8]. Therefore, an additional possibility that has gained increased attention is that exposure to excessed cortisol has been associated with the epidemic of CVD [9, 10]. Epidemiological and experimental evidence suggested that elevated cortisol was associated with increased insulin resistance (IR), decreased *ß* cell function, obesity, hyperlipidemia, etc. [11–14], which are the risk factors of CVD. Although existing research studies explored the associations between cortisol and CVD and CVD risk factors, the physiological role of cortisol in the prevalence of CVD among patients with T2DM is still unknown.

Vitamin D primarily mediates its effects by binding to vitamin D receptors (VDRs), which are widely expressed in many tissues and cell types. Vitamin D deficiency had been implicated in the incidence of CVD [15, 16]. In addition, a 6-month follow up randomized controlled study suggested that oral daily doses of vitamin D improve HbA1c levels over the 3-month and 6-month period [17]. However, results of clinical trials have shown little to no effect of vitamin D supplementation in preventing CVD risk factors or incidence [18, 19]. Cortisol and vitamin D are all derived from cholesterol metabolites. Clinical evidence had shown that elevated glucocorticoids use was associated with decreased serum 25 (OH)D levels among asthmatic children [20]. Additionally, experimental evidence has suggested that 1,25-dihydroxyvitamin D3 regulates adipocyte cortisol levels by increasing 11beta-HSD1 expression [21]. Thus, whether 25 (OH)D level affects the associations of cortisol with CVD and its risk factors requires further study.

Existing studies suggested that disrupted cortisol secretion may be associated with several cardiometabolic health problems, including T2DM, CVD, metabolic syndrome, etc. [9–11, 14]. With respect to CVD in particular, however, most previous studies have been limited to the elderly and women in developed countries [9, 10], and the related evidence among T2DM patients in rural regions is scant and inconclusive. In addition, as mentioned above, T2DM patients are twice as likely to develop CVD as healthy people. However, very limited such evidence on T2DM patients is available. Moreover, 25 (OH)D levels were shown to be negatively associated with CVD, but whether 25 (OH)D level has an effect on the associations of cortisol with CVD and its risk factors requires unknown. Therefore, we hypothesized that (1) serum cortisol concentrations were associated with CVD and its risk factors, and (2) 25 (OH)D might serve as a moderator in associations of serum cortisol with the prevalence of CVD and CVD risk factors. The evidence of both above would provide a reference for identifying and controlling cardiovascular risk factors among T2DM patients. The results of this study would provide potential interventions targeting the hypothalamic-pituitary-adrenal (HPA) axis and cortisol pathway in the prevention of CVD in T2DM patients. Meanwhile, the effect of 25 (OH)D on the associations of serum cortisol with CVD and CVD risk factors would set the stage for vitamin D intervention to prevent CVD risk among patients with T2DM.

## 2. Materials and Methods

### 2.1. Study Population

Participants in this study were from an ongoing prospective cohort conducted from July 2015 to September 2017 and recruited rural adults aged 17–79 years from Henan province, as reported previously [22]. To explore the associations of serum cortisol levels with CVD and its risk factors among patients with T2DM, we conducted a cross-sectional study including 917 patients diagnosed with T2DM, which were selected by using a simple random sampling method. Participants with missing information on serum cortisol (*n* = 49) or 25 (OH)D (*n* = 61), or important covariates (*n* = 45) were excluded. Therefore, our study included 762 patients with T2DM, and all of them signed informed consents. This study was approved by the Zhengzhou University Life Science Ethics Committee.

### 2.2. Definition of Outcomes

The definition of T2DM was met the American Diabetes Association (ADA) diagnostic criteria (2009), as a fasting plasma glucose (FPG)≥7.0 mmol/L, HbA1c ≥ 6.5%, or a self-reported history of T2DM and receiving hypoglycemic agents after excluding other specific types of diabetes. The definition of CHD and stroke was based on self-reported or New Rural Cooperative Medical System (NRCMS) medical records reviews, in which each subject had a unique medical insurance card number to track diseases. CHD and stroke as reviewed in NRCMS were defined by village doctors and further determined by a committee included an internist, an endocrinologist, a cardiologist, and an epidemiologist in accordance with World Health Organization criteria.

### 2.3. Covariates

Characteristics of study subjects were obtained by using a standard face-to-face questionnaire, including age, gender, marital status, average monthly individual income, physical activity, educational level, smoking and alcohol-consumption status, dietary habits, and family history of diseases. Smoking status was grouped into smokers (current and past smokers) or nonsmokers, and alcohol-consumption status was recorded as drinkers (current and past drinkers) or nondrinkers. Marital status was described as married/cohabitating or widowed/single/divorced/separation. Educational level was divided into elementary school or below, junior high school, and high school or above. Average monthly income was classified into three categories (>500, 500∼, and ≥1000 renminbi (RMB)). According to the International Physical Activity Questionnaire, physical activity was divided into three grades: low, medium, and high levels. Dietary habits included vegetables and fruits intake and fat diet were obtained by food frequency questionnaire (FFQ). The cutoff value of more vegetables and fruits intake or high fat diet was 500 g fruits and vegetables per day or 75 g livestock and poultry meat per day, respectively, according to the dietary guidelines for Chinese residents. The family history of T2DM was ascertained as parents of the respondents with a history of T2DM. Anthropometric measurements (height, weight, and waist circumference) were measured as previously described [22]. Three-seated blood pressure (BP) values were measured consecutively using an automated electronic (HEM-770A Fuzzy, Omron, Japan) after 5 min of rest, and the average values were taken for the final analysis. Body mass index (BMI) was calculated as weight (in kg)/height squared (in m). Pulse pressure (PP) was calculated as systolic blood pressure (SBP) minus diastolic blood pressure (DBP).

### 2.4. Laboratory Assessments

After at least 8h of overnight fasting, venous blood samples were collected from all participants at morning, and serum samples were stored at −80°C for the further analysis. FPG, total cholesterol (TC), triglycerides (TG), high-density lipoprotein cholesterol (HDL-C), and low-density lipoprotein cholesterol (LDL-C) were detected by using an automatic biochemical analyzer (ROCHE Cobas C501). HbA1c was measured with high performance liquid chromatography (VARIANT II, Bio-Rad, CA, USA). Fasting insulin was measured by the radioimmunoassay (GC-gamma radioimmunoassay counter, USTC ZONKIA, China). The calculation of HOMA2-IR and HOMA2-*β*% was based on a HOMA2 calculator downloaded from website to estimate IR and pancreatic *β*-cell function (https://www.dtu.ox.ac.uk/homacalculator/) [23]. The updated computer-based homeostasis model assessment (HOMA2) index was widely used in the current study due to nonlinear solutions [23, 24].

The concentrations of serum cortisol, 25 (OH)D_2_, and 25 (OH)D_3_ were measured with liquid chromatography-mass spectrometer system (LC-MS/MS) (a Waters XEVO TQ-S system (Waters, Milford, MA, USA)). Blank and quality control samples were performed on each batch of 12 samples to monitor the stability of the instrument. The limits of detection (LOD) were calculated at signal-to-noise (S/N) of 3. The LOD of cortisol, 25 (OH)D_2_, and 25 (OH)D_3_ were 0.2 ng/ml, 0.5 ng/ml, and 1.0 ng/ml, respectively. The concentrations of cortisol below the LOD were replaced by 1/2 LOD. 25 (OH)D was calculated as the sum of 25 (OH)D_2_ and 25 (OH)D_3_.

### 2.5. Statistical Analysis

#### 2.5.1. Preliminary Analyses

Continuous and categorical variables were shown as means (standard deviations (SD)) (normal distribution) or medians (interquartile ranges) (non-normal distribution) and numbers (percentages). Chi-square tests, *t*-tests, or Mann–Whitney *U* tests were used to compare the difference between groups for categorical, normal, and non-normal distribution continuous variables. Since the distributions of cortisol, 25 (OH)D, and all cardiovascular risk factors were skewed distribution, these indicators were ln-transformed to improve the normal distribution for the further analysis.

We used logistic and linear regression models to explore the associations of serum cortisol with the prevalence of CVD and its risk factors, with odds ratio (OR) or regression coefficient (*β*) and 95% confidence interval (CI) were reported. In the present study, three models were performed:

Model 1 Unadjusted.
Model 2 Adjusted for age and gender.
Model 3 Adjusted for age, gender, educational level, average monthly individual income, smoking status, alcohol consumption, physical activity, high fat diet, vegetables and fruits intake status, and family history of T2DM.

#### 2.5.2. Modification Analysis

We used SPSS PROCESS macro as the bootstrap method to test the hypothesis that 25 (OH)D moderates the associations between cortisol and CVD and its risk factors. PROCESS was performed using one independent variable (serum cortisol), one moderator (25 (OH)D), and one dependent variable (CVD or each cardiovascular risk factor). We used 5000 bootstrap samples in this analysis to determine the moderation effect of 25 (OH)D. Both the independent and moderator variables were mean centered before analysis. In addition, the concentrations of 25 (OH)D were divided into three levels: 1 SD below the mean, the mean, and 1 SD above the mean to examine conditional indirect effects. Furthermore, in order to visualize the moderation effects, we plotted the conditional effect to facilitate the interpretation of significant adjustments.

## 3. Results

### 3.1. Population Characteristics

The characteristics of the study population are shown in Table 1. The current study consisted of 286 males (37.53%) and 476 females (62.47%), with an average age of 59.86 years (data not shown). Subjects with CHD were more likely to have high levels of physical activity, PP, SBP, TG, and HOMA2-*β* than those with non-CHD (*P* < 0.05). In addition, compared with patients with stroke, participants with nonstroke tended to be younger, have lower values of PP and SBP, and have higher values of TC and LDL-C (*P* < 0.05). No other demographics or cardiovascular risk factors significantly differed between groups (*P* < 0.05).

### 3.2. Associations between Serum Cortisol Concentrations and CHD/Stroke

The associations between serum cortisol concentrations and CHD/stroke are summarized in Table 2. Each 1 SD increase in serum cortisol was associated with higher prevalence of stroke (OR: 1.22, 95% CI: 1.03, 1.45). This association was not attenuated after additional adjustment for confounders (OR: 1.25, 95% CI: 1.05, 1.50). Individuals in the third tertile of cortisol were not significantly associated with a higher prevalence of stroke compared with those in the first tertile (OR: 1.10, 95% CI: 0.68, 1.80). In addition, the associations between serum cortisol and CHD were no significant in both unadjusted and adjusted models.

### 3.3. Association between Serum Cortisol Concentrations and Multiple Cardiovascular Risk Factors

Associations of serum cortisol with cardiovascular risk factors are presented in Table 3. Every 100% increase in ln-transformed serum cortisol was associated with a 0.099 mmol/L (95% CI: 0.020, 0.178) increase in TG, a 0.029 mmol/L (95% CI: −0.044, −0.015) decrease in HDL-C, and a 0.063 mmol/L (95% CI: −0.107, −0.019) decrease in LDL-C after adjustment (Model 3). In addition, every 100% increment in ln-transformed serum cortisol was related to a 5.8% (95% CI: −8.7, −2.8) decrease in ln-transformed HOMA2-*β*, a 0.180 mmol/L (95% CI: 0.034, 0.326) increase in FPG, a 0.613 mmol/L (95% CI: −0.929, −0.296) decrease in fasting insulin, and a 0.099% (95% CI: 0.014, 0.184) increase in HbAlc in Model 1. Little change occurred when we further adjusted the model for educational level, average monthly individual income, smoking status, alcohol-consumption status, physical activity, vegetables and fruits intake status, and family history of T2DM.

### 3.4. Moderation Analysis

**Figure 1:**
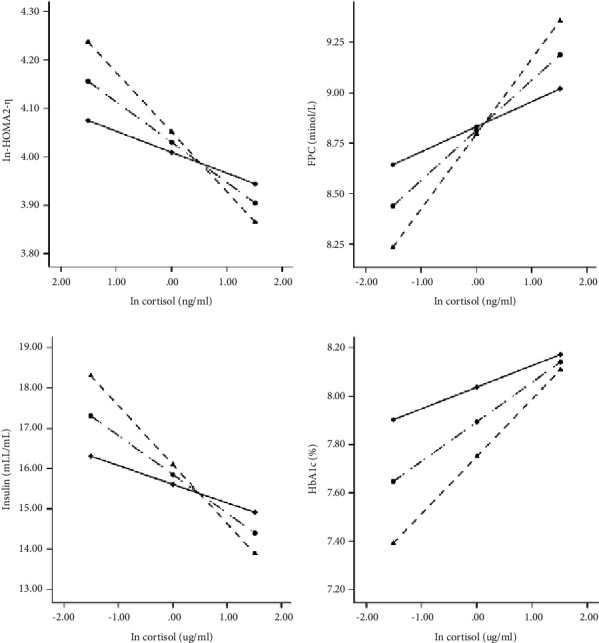
Associations between cortisol and cardiovascular metabolic risk factors for low, medium, and high levels of 25 (OH)D. The associations of cortisol and *ß* cell function, FPG, fasting insulin, and HbA1c moderated by 25 (OH)D were shown as (a), (b), (c), and (d), respectively. The model included the following covariates: age, gender, educational level, average monthly individual income, smoking status, alcohol consumption, physical activity, high fat diet, vegetables and fruits intake, and family history of T2DM. Δ: low levels; Ο: medium levels; ∇: high levels.

Associations between cortisol and CVD and its risk factors for low, medium, and high levels of 25 (OH)D are shown in Table 4. The conditional indirect effects on CVD and its risk factors were estimated at three levels of the 25 (OH)D (1 SD below the mean, the mean, and 1 SD above the mean) by the bootstrap method. As presented in Table 4, 25 (OH)D was found to be a significant moderator in the associations between cortisol concentrations and HOMA2-*β*, FPG, insulin, and HbAlc (*P* < 0.05), but not in the association between cortisol and CHD or stroke.  Figure 1 visualized these moderation effects by plotting simple slopes. Firstly, the effects of serum cortisol on HOMA2-*β* and fasting insulin were changed according to the range of 25 (OH)D level, indicating that subjects with high levels of serum cortisol and low levels of 25 (OH)D were more susceptible to have a lower state of HOMA2-*β* and fasting insulin than those with high levels of serum cortisol and 25 (OH)D (*P* < 0.05). Furthermore, the moderating effect of 25 (OH)D on FPG and HbA1c implied that individuals with high levels of serum cortisol and low levels of 25 (OH)D were more susceptible to have a higher state of FPG and HbA1c than those who exposed to high levels of serum cortisol and 25 (OH)D.

## 4. Discussion

This study was to examine the associations of serum cortisol with CVD and its risk factors (IR, *β* cell function, hyperlipidemia, obesity, etc.) in T2DM patients and to explore whether 25 (OH)D status modified the associations between serum cortisol and CVD or its risk factors. We confirmed that elevated levels of serum cortisol were associated with a higher prevalence of stroke and cardiovascular risk factors (deceased *ß* cell function, HDL-C, and fasting insulin as well as increased TG, LDL-C, FPG, and HbA1c). Furthermore, we found that the associations of serum cortisol with cardiovascular risk factors were modified by 25 (OH)D, suggesting that T2DM patients who had high levels of serum cortisol and low levels of 25 (OH)D were more susceptible to have higher levels of cardiovascular risk than those with high levels of serum cortisol and 25 (OH)D.

Our study found that elevated serum cortisol was associated with a higher prevalence of stroke, but not with CHD. Nevertheless, a population-based study suggested that higher hair cortisol was related to an increased prevalence of both stroke and CHD among community-dwelling elderly participants [25]. Findings from prospective cohort demonstrated a positive association between morning plasma cortisol and incident CVD, and evidence from the Mendelian randomization analyses was consistent with these multivariable results [10]. These findings suggested that elevated morning cortisol was a causal risk factor for CVD. In contrast, results from a large observational cohort study showed no evidence for the associations between hair cortisol and CHD diagnosis or the experience of a stroke incidence. Prior studies demonstrated the necessity of conducting studies in Chinese rural adults due to differential cortisol dynamics among various race/ethnicities, which could lead to differential outcomes.

Few previous studies have investigated the associations of cortisol and cardiometabolic risk factors in T2DM patients. One such study found that higher morning serum cortisol was associated with higher FPG and HbA1c in participants with diabetes [11], which is consistent with our findings. The relationships between cortisol and dyslipidemia have been reported in several previous studies, with inconsistent results. Stalder et al. found cortisol in relation to elevated TG and reduced HDL-C [8]. Our study illustrated that serum cortisol was associated with dyslipidemia (TC, TG, HDL-C, and LDL-C), impaired glycometabolism (*β* cell function, FPG, HbA1c, and fasting insulin) but not with SBP and DBP and weight-related indictors (BMI) in T2DM patients. Inconsistent with our study, a meta-analysis included 11 studies revealed that hair cortisol was associated with SBP, whereas the associations of cortisol with DBP were overall no significant [8]. Our present study population were T2DM patients, thus, long-term hyperglycemia may be cause differences results. We also found a negative association between cortisol and *ß* cell function, which is in line with Ortiz et al.'s study involving in participants without T2DM [11].

Although the mechanisms underlying the association between cortisol and CVD remain unclear, there are several plausible mechanisms. Cortisol is a major glucocorticoid that exerts physiological functions by activating glucocorticoid receptors, which are widely expressed in cells. A prior study has revealed that long-term elevated cortisol stimulates glucose production in the liver, and reduces glucose uptake and utilization by antagonizing insulin response in skeletal muscle and adipose tissue [26]. In addition, glucocorticoids regulate the secretion of glucagon and insulin by regulating the function of pancreatic *a* and *β* cells. Additionally, cortisol lead to dyslipidemia may attribute to direct and indirect cortisol action on lipolysis, free fatty acid production and turnover, very low-density lipoprotein synthesis, and fatty accumulation in the liver [27]. Further studies are warranted to definitively explore the potential mechanism of cortisol and cardiovascular risk factors.

At present, the association between vitamin D deficiency and CVD is still controversial, and its mechanism is still unclear. It may be related to the effect of renin angiotensin system, the elevation of blood glucose, the impact of inflammatory cytokines, and the direct impact on heart tissue and vascular [28]. Wang et al. found that the serum level of 25 (OH)D_3_ was negatively associated with carotid intima thickness in patients with T2DM, and low vitamin D status could promote the development of atherosclerosis in patients with T2DM [29]. Anastasi et al. showed that the level of 25 (OH)D of patients with myocardial infarction was significantly reduced, and vitamin D could be used in combination with other indicators such as troponin and brain natriuretic peptide to diagnose myocardial infarction [30]. A prospective study showed that coronary syndrome patients with low serum 25 (OH)D levels had a higher incidence of in-hospital complications and mortality [31]. Another study recruited 90 T2DM patients with serum 25 (OH)D levels less than 30 ng/ml, added 50000 IU vitamin D for eight weeks, and results showed a significant increase in serum vitamin D levels, decrease in serum HbA1c levels, and increase in HDL-cholesterol levels [32]. This experiment proved that vitamin D supplements can improve HbA1c and lipid metabolism in patients with T2DM. However, another study suggested that neither vitamin D nor vitamin D-specific receptor polymorphism has been proven to be associated with metabolic parameters and glycemic control in T2DM patients [33]. In addition, cortisol and 25 (OH)D have a shared substrate in cholesterol, and it has been hypothesized that the two compounds might compete for cholesterol as a substrate for synthesis [34]. Our study suggested that low 25 (OH)D level may alter the association between cortisol and CVD risk factors, suggesting that participants with high levels of serum cortisol and low levels of 25 (OH)D were more susceptible to cardiovascular and metabolic risk factors than those with high levels of serum cortisol and 25 (OH)D.

However, several limitations should be acknowledged in the present study. First, cortisol release following a strong circadian rhythm, but longitudinal measure of 24-hour urinary cortisol is challenging for a large population-based study. Second, since our study population is all T2DM patients, our findings have limited applicability to the general population. However, our current study suggested serum cortisol may be a reliable factor for the prevalence of CVD among T2DM patients, and similar associations were found in general population. Third, the design of this study was a cross-sectional study, which cannot establish causality inevitably. Fourth, this study found that serum cortisol was associated with stroke and cardiovascular risk factors. However, effects may be imprecisely estimated due to the small study sizes. Finally, although relevant variables were extensively adjusted, residual confounding factors remain in this study.

## 5. Conclusions

Elevated serum cortisol was associated with a higher prevalence of stroke and cardiovascular risk factors. 25 (OH)D might modify the associations between cortisol and cardiovascular risk factors, implying that subjects with high levels of serum cortisol and low levels of 25 (OH)D were more susceptible to have higher levels of cardiometabolic risk factors than those with high levels of serum cortisol and 25 (OH)D.

## Figures and Tables

**Table 1:** Basic characteristics of the study population.

Variables	Non-CHD (n = 690)	CHD (n = 72)	*P* value	Nonstroke (n = 649)	Stroke (n = 113)	*P* value
Males, n (%)	264 (38.26)	22 (30.56)	0.199	247 (38.06)	74 (65.49)	0.473
Age (y), mean (SD)	59.68 (8.74)	61.60 (7.65)	0.074	59.31 (8.72)	63.04 (7.53)	<0.001
Smoking status, n (%)			0.648			0.855
Current or past smoker	180 (26.09)	17 (23.61)		167 (25.73)	30 (26.55)	
Nonsmoker	510 (73.91)	55 (76.39)		482 (74.27)	83 (73.45)	
Alcohol consumption, n (%)			0.843			0.997
Current or past drinker	141 (20.43)	58 (80.56)		132 (20.34)	23 (20.35)	
Non-drinker	549 (79.57)	14 (19.44)		517 (79.66)	90 (79.65)	
Marital status, n (%)			0.169			0.031
Married/cohabitating	616 (89.28)	68 (94.44)		589 (90.76)	95 (84.07)	
Educational level, n (%)			0.760			0.158
Elementary school or below	386 (55.94)	39 (54.17)		356 (54.85)	69 (61.06)	
Junior high school	233 (33.77)	27 (37.5)		222 (34.21)	38 (33.63)	
High school or above	71 (10.29)	6 (8.33)		71 (10.94)	6 (5.31)	
Average monthly individual income, n (%)			0.939			0.390
<500, RMB	283 (41.01)	29 (40.28)		262 (40.37)	50 (44.25)	
500∼, RMB	188 (27.25)	21 (29.17)		184 (28.35)	25 (22.12)	
1000∼, RMB	219 (31.74)	22 (30.56)		203 (31.28)	38 (33.63)	
Physical activity, n (%)			0.006			0.035
Low	185 (26.81)	22 (30.56)		165 (25.42)	42 (37.17)	
Mediate	320 (46.38)	43 (59.72)		316 (48.69)	47 (41.59)	
High	185 (26.81)	7 (9.72)		168 (25.89)	24 (21.24)	
High fat diet (≥75 g/day), n (%)	122 (17.68)	11 (15.28)	0.609	120 (18.49)	13 (11.50)	0.071
Vegetables and fruits intake status (≥500 g/day), n (%)	444 (64.35)	42 (58.33)	0.312	413 (63.64)	73 (64.60)	0.844
Family history of T2DM, n (%)	36 (5.22)	4 (5.56)	0.785	35 (5.39)	5 (4.42)	0.670
BMI (kg/m^2^), median (IQR)	25.49 (4.26)	25.32 (4.10)	0.542	25.56 (4.10)	25.21 (5.22)	0.942
FPG (mmol/L), median (IQR)	7.89 (3.27)	7.59 (3.56)	0.060	7.87 (3.28)	7.90 (2.76)	0.358
Fasting insulin (uIU/mL), median (IQR)	14.12 (7.15)	14.21 (8.02)	0.841	14.23 (7.10)	13.70 (7.05)	0.994
HbA1c %, median (IQR)	7.40 (2.30)	7.35 (2.07)	0.866	7.40 (2.31)	7.30 (1.85)	0.590
PP (mmHg), median (IQR)	49.00 (15.00)	53.50 (19.17)	0.010	48.33 (1.47)	54.67 (62.67)	<0.001
SBP (mmHg), median (IQR)	125.00 (24.00)	129.50 (26.75)	0.021	124 (23.00)	133 (23.50)	<0.001
DBP (mmHg), median (IQR)	76.00 (15.000)	78.00 (13.00)	0.529	76.00 (14.00)	77.00 (14.00)	0.220
TC (mmol/L), median (IQR)	4.81 (1.24)	4.69 (2.08)	0.672	4.84 (1.30)	4.63 (1.37)	0.003
TG (mmol/L), median (IQR)	1.91 (1.64)	2.42 (2.05)	0.019	1.96 (1.74)	1.79 (1.50)	0.117
HDL-c (mmol/L), median (IQR)	1.23 (0.44)	1.21 (0.50)	0.213	1.23 (0.46)	1.24 (0.41)	0.781
LDL-c (mmol/L), median (IQR)	2.78 (1.17)	2.66 (1.66)	0.166	2.80 (1.22)	2.63 (1.34)	0.007
HOMA2-IR, median (IQR)	2.08 (1.09)	2.05 (1.15)	0.981	2.09 (1.10)	2.02 (1.08)	0.485
HOMA2-*β*%, median (IQR)	61 (47.58)	66.30 (64.65)	0.045	61.30 (50.60)	62.20 (40.25)	0.433
25 (OH)D (ng/ml), median (IQR)	29.89 (10.60)	28.59 (10.82)	0.547	29.75 (10.42)	30.16 (10.40)	0.290
Cortisol (ng/ml), median (IQR)	154.70 (96.25)	156.95 (94.88)	0.484	153.4 (94.85)	161.60 (115.05)	0.334

Data were presented as n (%) and mean ± SD or median (interquartile range) for categorical and continuous variables, respectively.

**Table 2:** Logistic regression analysis of associations between cortisol and CHD/stroke.

Variables	Logistic regression OR (95% CI)			
CHD	Per SD increment	T1	T2	T3
Model 1	1.12 (0.90, 1.39)	Reference	1.21 (0.66, 2.23)	1.27 (0.69, 2.31)
Model 2	1.15 (0.92, 1.42)	Reference	1.23 (0.67, 2.27)	1.29 (0.70, 2.36)
Model 3	1.12 (0.90, 1.39)	Reference	1.28 (0.69, 2.37)	1.28 (0.69, 2.35)
Stroke	Per SD increment	T1	T2	T3
Model 1	**1.22 (1.03, 1.45)**	Reference	0.79 (0.48, 1.32)	1.09 (0.68, 1.76)
Model 2	**1.28 (1.07, 1.53)**	Reference	0.77 (0.46, 1.29)	1.13 (0.70, 1.83)
Model 3	**1.25 (1.05, 1.50)**	Reference	0.78 0.47, 1.32)	1.10 (0.68, 1.80)

Significant association (*P* < 0.05) indicated by boldface type. Model 1: unadjusted. Model 2: adjusted for age and gender. Model 3: adjusted for age, gender, educational level, average monthly individual income, smoking status, alcohol consumption, physical activity, high fat diet, vegetables and fruits intake status, and family history of T2DM. In addition, significant association (*P* < 0.05) was indicated by boldface type. The tables that meet the requirements were in roman.

**Table 3:** Linear regression analysis of associations between cortisol and multiple cardiovascular risk factors.

Variables	Linear regression *β* (95% CI)		
	Model 1	Model 2	Model 3

HOMA2-*β*	**−0.058 (−0.087, −0.028)**	**−0.052 (−0.082, −0.023)**	**−0.052 (−0.082, −0.023)**
HOMA2-IR	−0.016 (−0.036, 0.004)	−0.015 (−0.035, 0.005)	−0.014 (−0.035, 0.006)
BMI	0.094 (−0.065, 0.253)	0.073 (−0.085, 0.231)	0.084 (−0.074, 0.242)
DBP	0.205 (−0.316, 0.727)	0.140 (−0.381, 0.661)	0.128 (−0.390, 0.646)
SBP	0.043 (−0.808, 0.894)	0.353 (−0.473, 1.180)	0.302 (−0.522, 1.125)
PP	−0.165 (−0.756, 0.427)	0.217 (−0.314, 0.749)	0.177 (−0.356, 0.711)
TC	−0.041 (−0.092, 0.011)	−0.035 (−0.085, 0.016)	−0.032 (−0.083, 0.019)
TG	**0.108 (0.028, 0.188)**	**0.094 (0.014, 0.173)**	**0.099 (0.020, 0.178)**
HDL-C	**−0.035 (−0.050, −0.019)**	**−0.030 (−0.045, −0.015)**	**−0.029 (−0.044, −0.015)**
LDL-C	**−0.072 (−0.116, −0.028)**	**−0.063 (−0.107, −0.020)**	**−0.063 (−0.107, −0.019)**
FPG	**0.180 (0.034, 0.326)**	**0.152 (0.007, 0.298)**	**0.159 (0.012, 0.305)**
Fasting insulin	**−0.613 (−0.929, −0.296)**	**−0.599 (−0.916, −0.282)**	**−0.579 (−0.897, −0.261)**
HbAlc	**0.099 (0.014, 0.184)**	**0.090 (0.004, 0.175)**	**0.093 (0.007, 0.179)**

Significant association (*P* < 0.05) indicated by boldface type. Model 1: unadjusted. Model 2: adjusted for age and gender. Model 3: adjusted for age, gender, educational level, average monthly individual income, smoking status, alcohol consumption, physical activity, high fat diet, vegetables and fruits intake status, and family history of T2DM. In addition, significant association (*P* < 0.05) indicated by boldface type. The tables that meet the requirements were in roman.

**Table 4:** Associations between cortisol and cardiovascular risk factors for low, medium, and high levels of 25 (OH)D.

Variables	Interaction effect	Lower 95% CI	Upper 95% CI	P for interaction

*CVD*				
CHD	−0.02082	−0.7070	0.2906	0.4133
Stroke	0.2042	−0.2272	0.6357	0.3536
*Cardiovascular risk factors*				
HOMA2-*β*	**−0.1498**	**−0.2427**	**−0.0569**	**0.0016**
TG	−0.0705	−0.3183	0.1773	0.5768
HDL-C	−0.0233	−0.0699	0.0233	0.3262
LDL-C	−0.0349	−0.1723	0.1026	0.6187
FPG	**0.4637**	**0.0044**	**0.9229**	**0.0478**
Fasting insulin	**−1.8685**	**−2.8580**	**−0.8791**	**0.0002**
HbAlc	**0.2767**	**0.0086**	**0.5449**	**0.0431**

Significant association (*P* < 0.05) indicated by boldface type. The moderation analyses were applied to identify whether 25 (OH)D altered the associations between serum cortisol and CVD and its risk factors. The model adjusted confounders of age, gender, educational level, average monthly individual income, smoking status, alcohol consumption, physical activity, high fat diet, vegetables and fruits intake, and family history of T2DM. Significant association (*P* < 0.05) was indicated by boldface type.

## Data Availability

The datasets and materials generated during and/or analyzed during the current study are available from the corresponding author on reasonable request.

## Abbreviations

T2DM: Type 2 diabetes mellitus
CVD: Cardiovascular diseases
OR: Odds ratio
CI: Confidence interval
FPG: Fasting plasma glucose
HbA1c: Glycated hemoglobin
IR: Insulin resistance
VDR: Vitamin D receptor
ADA: American Diabetes Association
CHD: Coronary heart disease
NRCMS: New Rural Cooperative Medical System
FFQ: Food frequency questionnaire
BP: Blood pressure
BMI: Body mass index
PP: Pulse pressure
SBP: Systolic blood pressure
DBP: Diastolic blood pressure
TC: Total cholesterol
TG: Triglyceride
HDL-C: High-density lipoprotein-cholesterol
LDL-C: Low-density lipoprotein-cholesterol
HOMA: Homeostasis model assessment
LC-MS/MS: Liquid chromatography-mass spectrometer system
LOD: The limits of detection
S/N: Signal-to-noise
SD: Standard deviation
HPA: Hypothalamic-pituitary-adrenal.
